# Left Ventricle: Fully Automated Segmentation Based on Spatiotemporal Continuity and Myocardium Information in Cine Cardiac Magnetic Resonance Imaging (LV-FAST)

**DOI:** 10.1155/2015/367583

**Published:** 2015-02-08

**Authors:** Lijia Wang, Mengchao Pei, Noel C. F. Codella, Minisha Kochar, Jonathan W. Weinsaft, Jianqi Li, Martin R. Prince, Yi Wang

**Affiliations:** ^1^Shanghai Key Laboratory of Magnetic Resonance and Department of Physics, East China Normal University, Shanghai, China; ^2^Department of Radiology, Weill Cornell Medical College, New York, NY 10022, USA; ^3^Multimedia Research, IBM T. J. Watson Research Center, 1101 Kitchawan Road, Yorktown Heights, NY 10598, USA; ^4^Department of Medicine-Cardiology, Weill Cornell Medical College, New York, NY 10021, USA; ^5^Department of Biomedical Engineering, Cornell University, Ithaca, NY 14853, USA; ^6^Department of Biomedical Engineering, Kyung Hee University, 1 Seocheon-dong, Giheung-gu, Yongin-si, Gyeonggi-do 446701, Republic of Korea

## Abstract

CMR quantification of LV chamber volumes typically and manually defines the basal-most LV, which adds processing time and user-dependence. This study developed an LV segmentation method that is fully automated based on the spatiotemporal continuity of the LV (LV-FAST). An iteratively decreasing threshold region growing approach was used first from the midventricle to the apex, until the LV area and shape discontinued, and then from midventricle to the base, until less than 50% of the myocardium circumference was observable. Region growth was constrained by LV spatiotemporal continuity to improve robustness of apical and basal segmentations. The LV-FAST method was compared with manual tracing on cardiac cine MRI data of 45 consecutive patients. Of the 45 patients, LV-FAST and manual selection identified the same apical slices at both ED and ES and the same basal slices at both ED and ES in 38, 38, 38, and 41 cases, respectively, and their measurements agreed within −1.6 ± 8.7 mL, −1.4 ± 7.8 mL, and 1.0 ± 5.8% for EDV, ESV, and EF, respectively. LV-FAST allowed LV volume-time course quantitatively measured within 3 seconds on a standard desktop computer, which is fast and accurate for processing the cine volumetric cardiac MRI data, and enables LV filling course quantification over the cardiac cycle.

## 1. Background

Quantifying left ventricular (LV) function is essential for diagnosing and planning therapy for cardiac diseases [1–3]. Modalities such as echocardiography, radionuclide cineangiography, multislice spiral computed tomography (CT), and cardiac magnetic resonance (CMR) imaging can be used to quantify LV volumes. CMR has the unique ability to provide anatomical images of the LV with both high temporal and spatial resolutions, without exposing the patient to ionizing radiation or contrast agents. CMR based volumetric measurements have been previously shown to be highly reproducible [4, 5] and are routinely used for the assessment of LV function [6] even during high-intensity exercise [7] or in heart disease [8]. More recently, diastolic function assessment based on LV volume has also been shown to be possible using CMR imaging methods [9, 10].

Traditional LV volume assessment has been limited to the examination of systolic performance using manual delineation of the LV endocardium at end systolic and end diastolic phases. It will take 5–10 minutes to manually delineate LV endocardium for a stack of about 20 images of the two phases [11]. Diastolic function assessment is still necessary and requires segmentation of the volume across all temporal phases. This results in upwards of 200 images requiring analysis, which is not practical for manual delineation. Recent diastolic functional evaluations are performed using dedicated software with manual segmentation of basal section within 25 minutes by an experienced radiologist [10–12]. Although there is evidence to support that the quantitative output for LV analysis between the commonly used software packages is not significantly different [13], the major variability of functional measurements derived from LV segmentation is strongly influenced by the visual selection of the basal slice and manual delineation of LV endocardium [8]. Thus, fully automated segmentation and quantification are necessary and of potential clinical utility for expanded applications of CMR in routine clinical practice.

Among existing automated methods [14], approaches with strong prior knowledge, such as active appearance model [15], Gaussian-mixture model [16], random walks [17, 18], and graph cuts [19–21], are ineffective for extracting accurate basal LV and excluding details like particular and trabecular mussels from LV. Approaches with weak or no prior knowledge, such as level sets [22], active contour model [23], iterative threshold-decreasing region growth [24] or multiseed region growth [25], and dynamic programming [26], typically require manual identification of the basal slices. In addition, approaches using long and short axis images also need manual intervention [27–29] to select the positions of the apex and mitral valves as well as the contour of long axis. Fully automatic LV segmentation has been difficult to achieve in practice. The commonly used cine SSFP acquisition of the heart includes slices beyond the apical and basal slices of LV, which are either manually excluded [24] or ignored altogether [25, 26, 30] in most of the automated segmentation. The basal slice often includes portions of the aortic root and is therefore not completely surrounded by ventricular myocardium, making it a challenge to automatically segment.

In this work we propose an LV segmentation method that addresses these challenges in a manner that is fully automated, based on the spatiotemporal continuity (LV-FAST). Using the assumption that cardiac volumes are smoothly varying through space and time, we can constrain the segmentation in each slice at a cardiac phase using information from spatiotemporally neighboring segmentations. This spatiotemporal continuity can be used to transfer knowledge from segmentations in areas of the LV that are easy to segment, for guiding segmentation in more challenging areas, such as the basal and apical slices, where previous methods have not been reliable and manual tracing is necessary.

## 2. Methods

### 2.1. Patients' Data and CMR Imaging Protocol

IRB approval was obtained for retrospective image analysis on deidentified data. This study involved 30 deidentified subjects who underwent Cine CMR, whose data was used for initial algorithm development and empirical parameter selection (Step 2 below). Additionally, 45 consecutive subjects (29 males, mean age 51.4 ± 18.8 years) who underwent Cine CMR exams between December 2010 and January 2011 were included in the study.  Table 1 details the characteristics of the 45 study population for quantitative analysis. This data was blind to analysis during algorithm development and used to evaluate algorithm performance.

Cine CMR image data were acquired using a 2D SSFP pulse sequence on 1.5 T (General Electric) scanners. In each subject, LV anatomy was imaged in 8–16 short axis slices from the level of the mitral valve annulus through the LV apex. Typical CMR imaging parameters were repetition time (TR) 3.5 ms, echo time (TE) 1.6 ms, flip angle 60°, matrix size 256 × 256, image dimensions 256 × 256, receiver bandwidth 125 kHz, FOV: 360 mm, slice thickness 6–8 mm, slice gap 2–4 mm (thickness + gap = 10 mm), and 20–28 reconstructed cardiac temporal phases.

### 2.2. Cine CMR Image Processing

Cine CMR image postprocessing, including initial LV localization and segmentation (henceforth referred to as Step 1) and apical and basal LV localization and segmentation (referred to as Step 2), was performed on a standard desktop computer with Intel Core 2 Duo 2.4 GHz processor and 2 GB RAM. Processing and execution times were assessed with the standard timer on the computer. Our fully automated method for quantitative LV analysis involved the following two segmentation stages.

**Step 1 (initial LV localization and segmentation).**

The middle slice of the imaged volume, which corresponds to the midventricular region of the heart, was used to determine an initial seed point for segmentation. This initial seed point was calculated by computing the difference image between the slice image from temporal phase 1 (nominal end diastole) and phase 8 (nominal end systole) and then extracting the center point of the difference image using the Hough transform [31]. By using the Hough transform of the difference image, we make sure that we capture a center point in the heart, which corresponds to the roundest object undergoing the most motion.

This seed point is then propagated across all temporal phases for the slice and used to segment those images. After segmentation across all images in this slice position, the center of mass of the LV blood pool in a segmented slice was used as the seed point on an adjacent slice in the superior and/or inferior directions.

A previously described iterative region growing method [24], which has been validated on prior subjects [11], was used for all segmentations carried out in Step 1 This method was repeated throughout all slices of the imaged volume and the results are subjected to further analysis in Step 2.

**Step 2 (estimate apical and basal slices from Step 1 using spatiotemporal continuity).**

**Figure 1:**
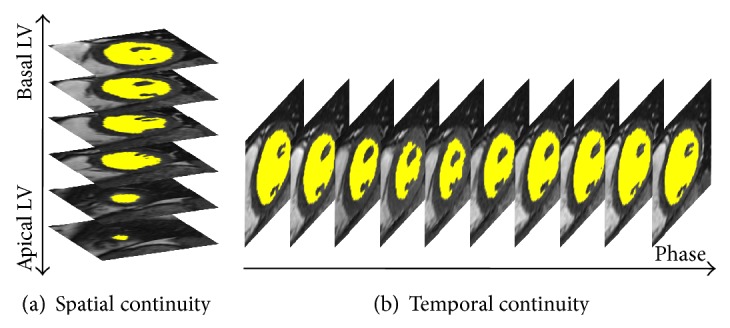
Spatiotemporal continuity of the LV section in cine MRI. (a) The short axis images from the apex to the base at a given cardiac phase depicting spatial continuity. The LV region (yellow) varies smoothly from one slice to next along the long axis. (b) Continuous cardiac phase images for a given slice depicting temporal continuity. The LV region (yellow) varies smoothly from one phase to the next along the time axis.

**Figure 2:**
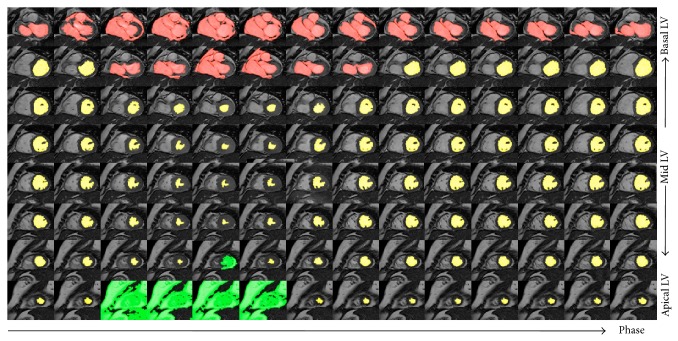
A simple example of the spatiotemporal continuity of the LV region from segmentation 0. The colored areas represent the LV region for various slices (vertical direction) at various phases (horizontal direction). The yellow region varies smoothly in slice and phase until LV is close to basal (red) and apical (green) slices.

**Figure 3:**
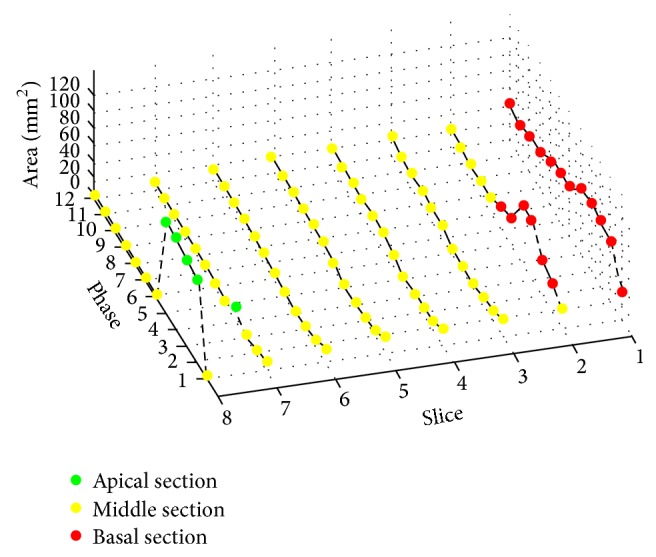
Circles represent the corresponding area of LV region shown in Figure 2. There are 8 slices and 12 phases, and the middle slice number is 4. The areas from slices 3 to 7 (yellow) vary smoothly in slice and phase axes, demonstrating spatiotemporal continuity. This continuity stops at slices 7 and 8 (green) and slices 1 and 2 (red), where there are abrupt increases in LV areas as Step 1 leaks out of the apex (slice 7) and basal slice (slice 2).

All short axis slices of the LV, except the most basal and apical segments, demonstrated volumetric spatiotemporal continuity: the LV volumes in these short axis slices were fairly smooth or changed slowly over the temporal cardiac phase and the slice location (shown in Figure 1). This continuity was terminated at the most basal and the apical slices, where the lack of myocardial encircling causes the segmentation algorithm in Step 1 to fail.  Figure 2 illustrates an example of the spatiotemporal continuity of LV region from Step 1. In Figure 3, the areas in red, yellow, and green represent the LV volume computed from Step 1. The areas with yellow were successful but with red and green color were failed in segmentation. In Figure 3, the *x*-, *y*-, and *z*-axes represent the slice, phase, and segmentation area, respectively, of the corresponding extracted region (shown in Figure 2). Each circle represents the segmented LV area of a slice at a corresponding phase. There are 8 slices and 12 phases. The middle slice was set to slice number 4 ( = 8/2). The areas from slices 3 to 7 (yellow) varied smoothly in slice and phase axes, demonstrating spatiotemporal continuity. This continuity stopped at slices 7 and 8 (green) and slices 1 and 2 (red), where there were abrupt increases in LV areas, as the algorithm of Step 1 leaked out of the apex (slice 7) and basal slice (slice 2). To characterize continuity, the images were ordered sequentially as in Step 1. *J* was defined as the ratio of the LV area of the segmented image of a given slice at a given phase, to the area of the LV of an adjacent slice at the same phase or the same slice at an adjacent phase. We referred to *J* as the “jump parameter.” We defined *D* as the corresponding mass center displacement between two images on adjacent slices at a given phase or the same slice at two adjacent phases, according to the geometric method [32]. If *J* > 2.5 or *D* > 6 pixels (empirically selected based upon 30 deidentified cases), the image was denoted as a “jump image,” and its corresponding phase and slice were denoted as a “jump phase” and “jump slice.” These jump images were close to the apex or base of the heart's LV region. Jump images denote where the algorithm of Step 1 likely failed, and further processing was necessary.

*(a) Apical Section Estimation*. When a “jump image” was detected in the apical end of the cardiac volume, a nearby phase was used to estimate the segmentation. We denote the estimated apical segmented area of a slice at phase *p* and slice *s* by *A*′(*p*, *s*). This value was computed from the LV area of slice *s* at the nearest phase *q* without a detected jump (successful Step 1 segmentation), denoted by *A*(*q*, *s*), using a linear scaling factor that represents the scale difference in volume of that section of the heart between the two phases:

(1) $$\begin{array}{c} A^{\prime }\left(p,s\right)=A\left(q,s\right)*\frac{\sum_{i=\text{ms}}^{s-1}A\left(p,i\right)}{\sum_{i=\text{ms}}^{s-1}A\left(q,i\right)}, \end{array}$$

where “ms” is the middle slice of the imaged volume. If the number of nonjump phases at slice *s* was large (at least 90%), the corresponding phases at slice *s* + 1 were segmented according to Step 1, and the apical areas of slice *s* + 1 at various phases were calculated using a cone interpolation. For any phase *p*, the apical area of slice *s* is related to that of its proceeding two adjacent slices *s* − 1 and *s* − 2 according to

(2) A′p,s=4Ap,s−1+Ap,s−2 −4Ap,s−1×Ap,s−2.

**Figure 4:**
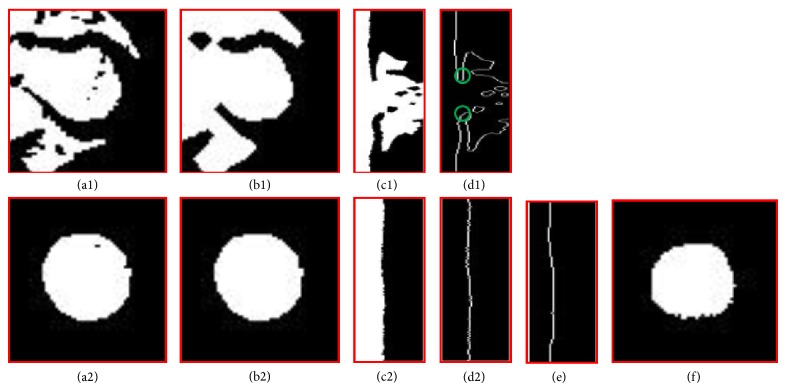
Basal image position and LV region estimation. (a1) and (a2) are binary images extracted using successive decreasing threshold based region growing. (b1) and (b2) show morphology processing on (a1) and (a2), respectively; (c1) and (c2) represent the circle mapping on (b1) and (b2), respectively; (d1) and (d2) are the edges of (c1) and (c2), respectively; (e) is line fitting and (d1) is the LV region transformed from polar to Cartesian coordinate based on (f).

*(b) Basal Section Segmentation*. The jump parameters were used to locate basal slices. In accordance with previously established conventions, the most basal LV image has at least 50% of its LV circumference in contact with myocardium [33]. The percentage of this circumferential myocardium was estimated by identifying the myocardial termination points using the denoising and identification steps as illustrated in Figure 4. At a given phase, the first jump slice from Step 1 and the slice immediately prior are shown in Figures 4(a1) and 4(a2). The noisy figures in the LV masks in these two slices were then smoothed by applying a morphology closing and then opening with a disk of 3 pixel radius (Figures 4(b1) and 4(b2)). The apparent myocardial border point and therefore its radius at a given angle were used as the first zero point in the radial direction in the polar map of the LV masks generated by Step 1 (Figures 4(c1), 4(d1), 4(c2), and 4(d2)). The two myocardial termination points were determined in the polar map. The two pixels immediately beyond the apparent myocardial border were labeled as apparent myocardial points (green circles in Figure 4(d1)). The zero angle line was set to the middle of the region of the jump slice where the difference between the apparent myocardial radius and the last nonjump slice was less than *D* pixels. The two apparent myocardial termination angle lines were then searched from zero angles in the top to the 360° angle line in the bottom in Figure 4(d1) as the first angles where the apparent myocardial radius abruptly increased by more than *D* pixels compared to those in Figure 4(d2). The jump slice was identified as not containing the LV if the average intensity of the apparent myocardial points in the jump slice differed from that of the last nonjump slice by more than 30% (to rule out atrium or aorta) or if the angle extending past the apparent myocardium was less than 180° (50% circumferential myocardium in the definition of the most basal slice). In this manner, the two myocardial terminal points were identified, and a straight line between them in the Cartesian coordinate was used to define the LV region as in manual segmentation of the basal slice done by a clinician.

The main parameters mentioned in Step 2 were summarized in Table 2.

### 2.3. Experiment

The LV-FAST algorithm was applied to cardiac short axis cine SSFP CMR images in order to obtain the following LV parameters: end diastolic volume (EDV), end systolic volume (ESV), ejection fraction (EF), volume filling curves, and the most basal and apical slices at each temporal phase. The LV-FAST derived parameters (EDV, ESV, and EF) were compared with the LV-METRIC algorithm [24] that was used by experienced operators (MK) with the following manual interventions. (1) The most basal and apical slices were visually inspected and identified. (2) The basal LV region was manually segmented. The LV-METRIC algorithm was implemented on a workstation (Advantage; GE Healthcare) with an Intel Xeon 3.4-GHz processor and 4 GB of random access memory.

### 2.4. Statistical Analysis

The mean and standard deviation of paired differences (*P* < 0.05) were calculated to compare the automated and manual results in this study. A linear regression was performed to assess correlations between the manual and fully automated measurements, and a Bland-Altman analysis was used to compare volume estimations between manual tracing and the automated methods.

## 3. Results

**Figure 5:**
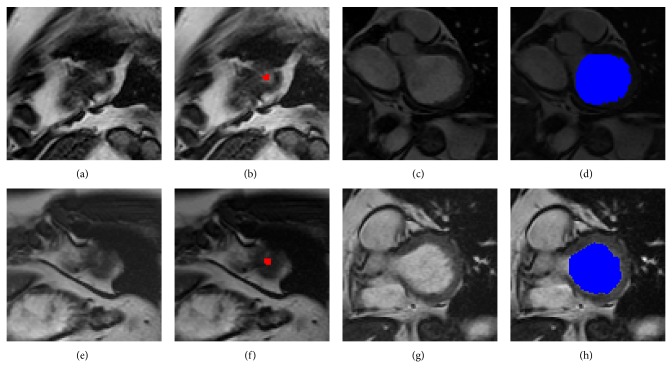
Two typical examples of the most apical slice estimation and the most basal slice segmentation. (a) and (e) are the original apical image; (b) and (f) are the corresponding areas estimated by LV-FAST. (c) and (g) are the original basal image; (d) and (h) are the corresponding segmentation results by LV-FAST.

**Figure 6:**
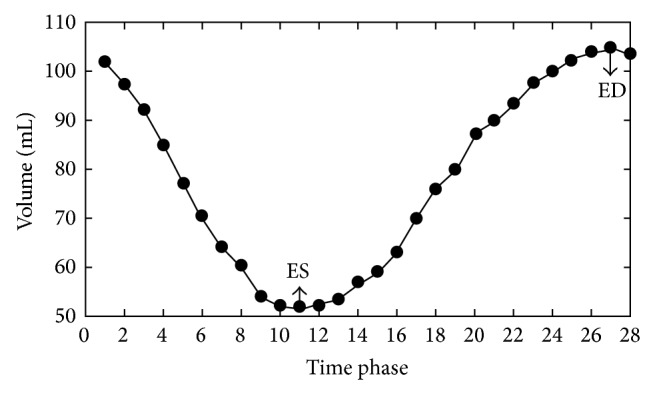
An example of the filling curve from fully automated segmentation. It shows the volume (vertical axis) change of the LV along phase time (horizontal axis).

A smooth spatiotemporal area map was acquired for quantifying LV.  Figure 5 shows two typical examples of the most apical slice estimation and the most basal slice segmentation. Figures 5(a) and 5(e) show the original apical image; Figures 5(b) (red) and 5(f) (red) show the corresponding areas estimated by LV-FAST. Figures 5(c) and 5(g) show the original basal image; Figures 5(d) (blue) and 5(h) (blue) show the corresponding segmentation results by LV-FAST.  Figure 6 illustrates a typical example of endocardial volume versus cardiac phase; analysis was performed in less than 3 seconds.

Of the 45 consecutive cases, LV-FAST and manual segmentation agreed in over three-fourths of cases: The two approaches detected the identical apical slices at ED and ES phases in 84% (*n* = 38) and 91% (*n* = 41) of cases. For basal slice identification, the two approaches agreed 84% (*n* = 38, 38) cases for both ED and ES. For the 7 cases in which automated and manual results did not match for defining the most apical slice at ED, LV-FAST overestimated one slice in 4 cases and underestimated one slice in the remaining 3 cases. For all 4 discrepant cases in defining the most apical position at ES, LV-FAST overestimated one slice in all 4 cases. For the 7 discrepant cases in defining the most basal slice at ED, LV-FAST overestimated one slice in 3 cases and underestimated one slice in the remaining 4 cases. Finally in the 7 discrepant cases in defining the most basal slice at ES, LV-FAST overestimated one slice in 4 cases and overestimated two slices in the remaining 3 cases.

**Figure 7:**
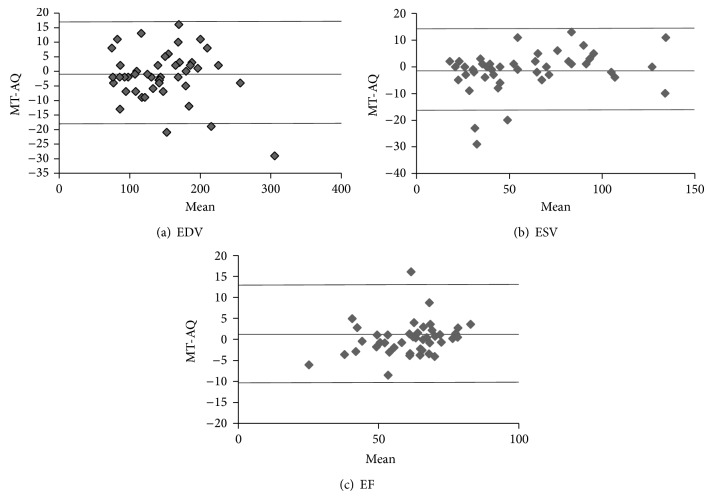
Bland-Altman analyses show results of the comparison between fully automatic quantification (AQ) and manual tracing (MT) in EDV, ESV, and EF. The comparisons of EDV, ESV, and EF for all the 45 cases between AQ and MT are shown with these plots in (a)–(c).

LV volumes at ED, ES, and EF measured by the fully automated LV-FAST and manual segmentation are summarized in Table 3. In the 45 consecutively selected patients, LVEDV measured by LV-FAST and manual segmentation were 148 ± 52 mL and 146 ± 50 mL, respectively (*P* = 0.41); mean volumes in ES were 60 ± 31 mL and 58 ± 33 mL, respectively (*P* = 0.29); EF were 61 ± 12% and 62 ± 13%, respectively (*P* = 0.25). Corresponding Bland-Altman plots for between automated LV-FAST and manual segmentation results are illustrated in Figure 7. The means ± standard deviations of manual measurement minus automated measurement across all subjects were −1.6 ± 8.7, −1.4 ± 7.8, and 1.0 ± 5.8 for EDV, ESV, and EF, respectively. Of note, there were two outliers in EDV (Figure 7(a)), in which one was caused by the area overestimation at the most basal slice and the another by the slice overestimation at the most apical slice; there were three outliners in ESV: one was caused by the area overestimation and two were caused by the slice overestimation at the most basal slice (Figure 7(b)). There was one outliner in EF, which was caused by the slice overestimation at the most basal slice (Figure 7(c)).

## 4. Discussion

We proposed a fully automated method for quantifying the LV functional parameters using spatiotemporal continuity (LV-FAST), which can define the basal and apical slices at all cardiac phases and estimate corresponding LV volumes. Our experimental results on LV volume measurements showed that LV-FAST is in good agreement with the manual segmentation results by an experienced physician, suggesting that LV-FAST can be used for fast quantification of the LV stroke volumes, ejection fractions, and enabling filling curves on routine clinical CMR that is not possible with manual segmentation.

Unlike prior automated algorithms [12, 15–23, 25–30], this LV-FAST algorithm makes use of the spatiotemporal continuity of the 4D (cine volumetric) LV data to define the apex and base automatically and estimate the LV volume. The LV-FAST method starts with the midventricular slice using the iterative decreasing threshold algorithm, whose accuracy and robustness in clinical use are proven in [11]. The LV-FAST method then progresses to the next slice towards the apex until the area segmented at a slice jumps at a phase. The areas of last two slices may be corrected with temporal or spatial interpolation. However, because the area of the most apical slice is small, there is almost no effect on the quantitative analysis. After completing the estimation of the most apical slice, LV-FAST progresses from the midventricular slice to the basal section slice. The position of the basal slice is also defined by the area spatiotemporal continuity with the corresponding basal area estimated using a spatial or temporal shape constrained iterative region growing after calculating percentage or intensity of the circumferential myocardium.

There are differences between LV-FAST and manual segmentation, as described in Section 3. The discrepancies at the apex minimally affected calculated chamber volumes, whereas basal discrepancies yielded larger volumetric differences. Regarding this issue, it is important to recognize that, for LV-FAST, “jump” detection for defining the basal slice, *J* > 2.5, or *D* > 6 empirical values was based upon a learning algorithm generated from 30 CMR exams, which may not be suitable for all cases. In our 45 consecutively selected subjects, two subjects were found with large shifts between slices. This large slice misregistration might be caused by inconsistency in breath-holding or cardiac arrhythmia, both of which could be causal-FAST's misdetection of the basal slice. Estimation of myocardial circumference might be affected by the low image contrast and signal-to-noise ratio close to the most basal slice. Image quality improvement for Cine CMR such as 3D cine imaging [34] under free breathing is needed to eliminate artifacts such as slice misregistration caused by multiple breath holds used in acquiring multiple slices in current 2D cine cardiac CMR, which would preserve the physical spatiotemporal continuity and would improve the performance of LV-FAST.

## 5. Conclusion

In summary, an algorithm for left ventricle segmentation with full automation using spatiotemporal continuity (LV-FAST) is presented for fast and accurate processing of the cine volumetric cardiac MRI data, enabling LV filling course quantification over the cardiac cycle.

## Figures and Tables

**Table 1:** Characteristics of the 45 subjects.

Age (mean ± std)	51.4 ± 18.8
Male gender	64% (29)
Hypertension	40% (18)
Hyperlipidemia	36% (16)
Diabetes mellitus	20% (9)
Tobacco use	11% (5)
Family history	8% (4)

Note: data are percentages with numbers of subjects in parentheses.

**Table 2:** Summarization of main parameters.

Parameter	Value	Discussion
Middle slice (ms)	$\frac{\text{total slice}}{\text{2}}$	No influence on the algorithm

Jump factor (*J*)	*J* > 2.5	(1) Less than 2.5: LV volume will be underestimated. (2) More than 2.5: LV volume will be overestimated.

Mass center displacement (*D*)	*D* > 6 pixels	(1) Less than 6: the slice with close myocardium is defined as the basal slice, which will result in the underestimated LV volume. (2) More than 6: the basal slice without close myocardium cannot be detected, which will result in the overestimated LV volume.

Note: “*J*” and “*D*” are empirically parameters upon 30 deidentified cases.

**Table 3:** Results of quantification in all 45 subjects from LV-FAST and manual segmentation (MS).

Parameters	*n* = 45	
	MS	LV-FAST
EDV (mL)	146.2 ± 50.0	147.8 ± 51.7
ESV (mL)	58.1 ± 32.8	59.5 ± 30.6
EF (%)	61.9 ± 13.3	60.8 ± 11.7

Note.

(i) Unless otherwise specified, data are means ± standard deviations across all subjects in each group.

(ii) No statistical significance was found between MS and LV-FAST.

(iii) In the 45 consecutively selected subjects, corresponding *P* values to ED, EV, and EF were 0.41, 0.29, and 0.25, respectively.

## Abbreviations

EF: Ejection fraction
LV: Left ventricle
ED: End diastole
ES: End systole
CMR: Cardiac magnetic resonance
SSFP: Steady state free precession
EDV: End diastolic volume
ESV: End systolic volume.
